# Cost-Effectiveness in Patients Undergoing Revascularization of Chronic Total Occluded Coronary Arteries—A Cohort Study

**DOI:** 10.3389/fcvm.2022.849942

**Published:** 2022-05-26

**Authors:** Emil Nielsen Holck, Naja Stausholm Winther, Lone Juul Hune Mogensen, Evald Høj Christiansen

**Affiliations:** ^1^Department of Cardiology, Aarhus University Hospital, Aarhus, Denmark; ^2^Department of Clinical Medicine, Aarhus University, Aarhus, Denmark

**Keywords:** chronic total occlusion (CTO), chronic coronary syndrome (CCS), ischemic heart disease, complex PCI, coronary artery disease, percutaneous coronary intervention (PCI)

## Abstract

**Background:**

Revascularization of patients with chronic total occluded coronary arteries (CTO) is recommended if they have symptoms despite medical treatment. The cost-effectiveness of treatment with percutaneous coronary intervention (PCI) was investigated in this cohort study.

**Materials and Methods:**

The study was designed as a cohort study enrolling all patients undergoing PCI for a CTO in the Central Region of Denmark and recorded in the EUROCTO database. Major adverse cardio- and cerebrovascular events (MACCE) and admissions for cardiac symptoms were collected in the Western Denmark Heart Registry and through medical Journal Audits. Exposure was defined as successful revascularization of all CTO lesions compared with having one or more remaining CTOs after PCI attempt(s). Cost-effectiveness was evaluated as the net benefit (NB) at the patient level 3 years after treatment and through cost-effectiveness planes. The cost was defined as the cumulative cost of the index procedure and admissions due to MACCE and cardiac symptoms. Effectiveness was defined as the difference in MACCE for the primary analysis and the difference in death and symptomatic admissions for the secondary.

**Results:**

Between 2009 and 2019, 441 patients with ≥ 3 years of follow-up were treated with PCI for at least one CTO lesion (342 in the successful arm and 99 in the unsuccessful arm). The technical success rate was 85.4%. In total, 155 MACCE and 184 symptomatic admissions occurred in the follow-up period. The mean total cost was EUR 11.719 (11.034; 12.406) in the successful group vs. EUR 13.565 (11.899; 15,231) (*p* = 0.02) in the unsuccessful group. Net-benefit was EUR 1.846 (64; 3,627) after successful revascularization for MACCE. The adjusted analysis found an NB of EUR 1,481 (–118; 3,079). Bootstrap estimates showed cost-effectiveness planes in favor of successful revascularization.

**Conclusion:**

Patients fully revascularized for all CTO lesions had a more cost-efficient treatment. However, results need confirmation in a randomized controlled trial due to the risk of residual confounding after adjustment.

## Introduction

Revascularization of patients with chronic total occluded coronary artery (CTO) lesions is recommended in patients with angina resistant to medical treatment and/or large ischemic burden (1). The decision to perform percutaneous coronary intervention (PCI) should always balance the risks and benefits. Adequately powered trials have shown an improvement in the quality of life after PCI (2) and prognostic benefit has been indicated in observational studies (3). However, a recent meta-analysis of prospective randomized trials did not confirm an improvement in prognosis (4). The complexity of CTO lesions is leading to increasing procedural complication rates (5, 6). Due to an increasing burden of coronary heart disease worldwide (7), economic considerations need to be taken into account when selecting a treatment strategy. In the ORBITA trial, the investigators found that the cost per gained quality-adjusted life-years for a cohort of 1,000 patients was £ 90,218 (8). In a CTO population, the cost-effectiveness of PCI treatment is of particular interest due to more and longer procedures with the usage of several dedicated utensils (9). However, patients with untreated CTO lesions often have more symptoms and a more complex disease profile (10, 11) and therefore probably more hospital admissions. The current study aimed to investigate the difference in cost for the index procedure and subsequent admissions due to heart disease in patients who had been successful or unsuccessfully treated for their CTO lesions 3 years after the index procedure. The hypothesis was that patients who were successfully treated for their CTO had a more cost-effective treatment at follow-up. The following article is reported according to Strengthening the Reporting of Observational studies in Epidemiology (STROBE) statement.

## Materials and Methods

### Design and Study Population

The current study was conducted as an observational cohort study. The exposure of interest was successful vs. unsuccessful revascularization of all CTO lesions. Patients were eligible for enrollment if they were registered in the EUROCTO database, underwent CTO PCI at Aarhus University Hospital, and were citizens of the Central Region of Denmark at the time of event audit. All consecutive patients enrolled in the EUROCTO database who were treated at Aarhus University Hospital from 1 January 2009 to 31 December 2019 were entered into the study registry and merged with follow-up data on cardiac events. All patients who had completed 3 years follow-up were enrolled in the present study. During the enrollment period, Aarhus University Hospital was the only PCI center in the entire Central Region of Denmark (1.3 million inhabitants) performing approximately 100–120 CTO PCI cases a year. Non-CTO lesions were treated according to guidelines if technically feasible and safe, before treating CTO-lesions.

### Exposure

Successful treatment was defined as no persisting CTO-lesions after an attempt to open all CTO’s. An attempt to open a CTO could include one or more staged procedures at the operator’s discretion. Unsuccessful was defined as failure to open index CTO and/or other bystander CTO-lesions after the final attempt. Bystander CTO-lesions were left untreated by the decision of the treating heart team or physician.

### Outcome

All major adverse cardio- and cerebrovascular events (MACCE) during the index hospitalization or requiring hospital admission were registered through the entire follow-up period of 3 years. MACCE included all-cause death, myocardial infarction, stroke, target vessel failure, and decompensated chronic heart failure. Furthermore, all subsequent acute admissions where patients were discharged with an ICD-diagnosis describing that they had been admitted to observation for acute coronary syndrome (ACS) were registered. Outcome data were collected through medical record audits and from the Western Denmark Heart Registry (WDH) (12). All entries into the EUROCTO database, WDH, and medical records are performed consecutively in clinical practice at the time of procedure or event, meaning that data entry was prospective for all admissions and out-patient visits at all five acute hospitals in the Central Region of Denmark. Event audit was performed between 1 February 2020 and 1 December 2020. Events were adjudicated by Naja Stausholm Winther and Emil Nielsen Holck.

The primary endpoint in the current study was cumulative cost (net benefit (NB)) at patient-level 3 years after index CTO treatment between the successful (no remaining CTO’s) and unsuccessful (one or more remaining CTO’s) revascularization. The secondary endpoints were the difference in procedural cost and the cost per patient-year of follow-up. Furthermore, the incremental cost-effectiveness ratio (ICER) was calculated to assess the cost-effectiveness and plotted in a cost-effectiveness plane (CE-plane). The CE-planes were made with three effectiveness parameters: risk difference at 3 years for (1) death, (2) MACCE, and (3) suspected ACS. Cost calculation in the primary analysis was calculated using a Danish nationwide tool to group patients in 958 (In 2021) different cost categories (DRG) that have been used since 2004 to financially manage the entire public and private healthcare sector. The “DRG-rate” includes the average total cost for an admission in a specific disease category that year. The grouping for a complex PCI procedure was 05MP38 in 2021, with an average cost of EUR 5,037.72. The cost for index CTO treatment was calculated as the number of procedures multiplied by the 2021 DRG-rate added to the total cost of dedicated CTO equipment used at the index procedures and the DRG-rate of in-hospital complications, in case of complications. After discharge, the cumulative cost of all admissions due to cardiac disease or cardiac symptoms was calculated by adding the DRG-rate of these admissions. The primary analysis used the cumulation of costs from index procedure and event costs.

### Statistics

Continuous variables are given as means ± SD or medians [interquartile range, IQR] depending on distribution, while frequencies are represented by *n* (%). No statistical testing is performed for descriptive statistics due to adherence to the STROBE statement (13). The confidence interval (*CI*) of the main endpoint, NB, is calculated by non-parametric bootstrapping of the observed values. Survival curves for cumulative incidence of death, MACCE, and suspected ACS are plotted using Kaplan–Meier estimates for death and Nielson-Aalen estimates accounting for multiple events for MACCE and suspected ACS. ICER was calculated with both the mean and median cost difference in the numerator and the risk difference at 3 years in the denominator. In addition, 5,000 non-parametric bootstrap estimates for cost-effectiveness pairs were calculated and plotted in the CE-plane and elliptic 95% *CI*s were calculated using the lower 2.5% and upper 97.5% limits of the bootstrap estimates. Adjusted analysis of NB was performed with multiple linear regression, adjusting for age, sex, left ventricular ejection fraction (LVEF), three-vessel-disease, and chronic kidney disease since previous analysis have found these to be independent predictors of MACCE in the investigated cohort after performing a backward elimination model. A sensitivity analysis investigating bootstrapping of median values in the CE-plane and the difference in NB in successful vs. unsuccessful revascularization of the target CTO only was performed. The sample size was the total number of possible enrollments during the study period.

## Results

### Baseline Characteristics

**FIGURE 1:**
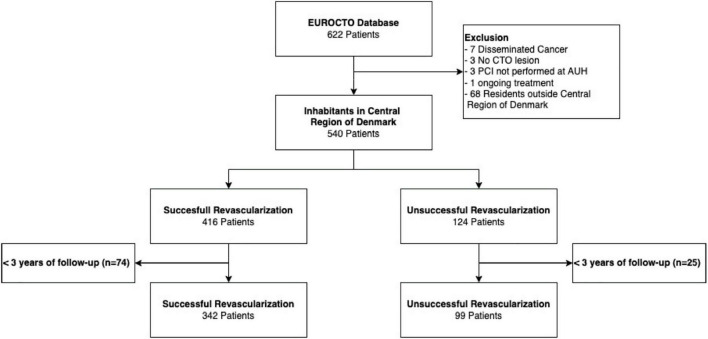
Flowchart.

The main population in the current study consists of 441 patients with residence in the central region of Denmark. Of these, 342 patients had all CTOs opened (successful group) and 99 had one or more remaining CTO’s (unsuccessful group). In total, 622 patients were identified in the EUROCTO database. The reasons for not being included in the primary analysis were mostly not residing in the Central Region of Denmark and < 3 years of follow-up (Figure 1). Patients with successful recanalization had an overall lower frequency of risk factors compared to patients with remaining CTOs. More patients had three-vessel disease, a lower LVEF, and a higher s-creatinine in the unsuccessful group. The Charlson comorbidity index was 3.3 ± 1.8 in the successful vs. 4.0 ± 1.7 in the unsuccessful group (Table 1).

**TABLE 1:** Baseline characteristics.

	Successful (*n* = 342)	Unsuccessful (*n* = 99)
Age	64.7 ± 10.8	67.8 ± 11.2
Sex	70 (20.5%)	19 (19.2%)
Familiar heart disease	147 (43.1%)	54 (54.5%)
Hypertension	209 (61.5%)	66 (67.3%)
Dyslipidemia	266 (78.0%)	82 (82.8%)
Peripheral disease	25 (7.4%)	11 (11.2%)
Diabetes mellitus		
Non-insulin dependent	60 (17.8%)	14 (14.4%)
Insulin dependent	22 (6.5%)	10 (10.3%)
BMI	28.4 ± 5.2	28.0 ± 4.6
Smoking		
Previous smoker	152 (46.8%)	54 (56.8%)
Active smoker	100 (30.8%)	17 (17.9%)
Previous CABG	51 (14.9%)	20 (20.2%)
Previous PCI	186 (54.4%)	63 (63.6%)
Previous MI	123 (36.0%)	39 (39.4%)
Left ventricular ejection fraction	52.8 ± 11.7	50.1 ± 12.3
Creatinine	93 ± 54	106 ± 92
Charlson comorbidity index	3.3 ± 1.8	4.0 ± 1.7
CCS class		
I	57 (16.7%)	24 (24.5%)
II	238 (69.6%)	50 (51.0%)
III	28 (8.2%)	15 (15.3%)
IV	4 (1.2%)	3 (3.1%)
Indication		
Chronic coronary syndrome	293 (85.7%)	81 (81.2%)
Acute coronary syndrome	39 (11.4%)	14 (14.1%)
Number of diseased vessels (including CTO)		
One-vessel	159 (46.5%)	20 (20.4%)
Two-vessel	112 (32.7%)	37 (37.8%)
Three-vessel	71 (20.8%)	41 (41.8%)
CTO vessel		
RCA	198 (57.9%)	46 (46.5%)
LAD	94 (27.5%)	25 (25.3%)
LCx	48 (14.0%)	28 (28.3%)
LM	2 (0.6%)	0 (0.0%)
JCTO score	3.1 ± 1.2	3.4 ± 1.0
Residual syntax	2.4 ± 9.3	15.5 ± 12.2

*BMI, body mass index; CABG, coronary artery bypass grafting; PCI, percutaneous coronary intervention, MI, myocardial infarction, CCS, Canadian cardiovascular society; CTO, chronic total occlusion; RCA, right coronary artery; LAD, left anterior descending; LCx, left circumflex; LM, left main. Numbers are given as n (%) or mean ± SD.*

### Procedural Characteristics

The mean number of attempts were 1.4 ± 0.6 in the successful group vs. 1.5 ± 0.8 in the unsuccessful group, and the mean number of persisting CTO’s were 1.1 ± 0.3 in the unsuccessful group. The overall technical success rate (i.e., successful index CTO-lesion treatment) for the entire cohort was 85.4%. Fewer balloons and stents were used in the unsuccessful group. Table 2 shows the CTO equipment used for cost calculations. In the successful arm, 6.7% of patients had an in-hospital complication requiring additional treatment, and for the unsuccessful group this fraction was 7.1%, primarily driven by a large fraction of acute renal failure (3.8 vs. 4.0%) (Supplementary Table 1).

**TABLE 2:** Procedural utensils and baseline medication.

	Successful (*n* = 342)	Unsuccessful (*n* = 99)
**Procedural data**		
Attempts	1.4 ± 0.6	1.5 ± 0.8
Number of persisting CTO’s	NA	1.1 ± 0.3
Number of guidewires used	5.2 ± 3.8	5.8 ± 2.9
Number of balloons used	4.5 ± 2.8	3.1 ± 2.8
Numbers of stents placed	2.3 ± 4.3	1.2 ± 1.5
Total stent length	58.2 ± 33.9	30.3 ± 41.8
IVUS used	54 (15.8%)	8 (8.1%)
Number of microcatheters used	1.3 ± 0.9	1.2 ± 0.9
Rotablation used	9 (2.6%)	1 (1.0%)
Guide extension used	44 (12.9%)	11 (11.1%)
Procedure length (minutes)	83.2 ± 52.8	87.0 ± 50.5
Contrast used (mL)	182.6 ± 90.4	192.5 ± 92.8
Cumulative Air Kerma (mGy)	1,359 ± 1,413	1,552 ± 1,326
Dose area product (CGY*cm^2^)	6,582 ± 8839.9	6,370 ± 9,005
**Successful strategy**		
Antegrade wiring	219 (64.0%)	NA
Antegrade dissection and re-entry	49 (14.3%)	NA
Retrograde wiring	15 (4.4%)	NA
Retrograde dissection and re-entry	56 (16.4%)	NA
**Medication use**		
Statins	319 (93.3%)	89 (89.9%)
Other lipid lowering drugs	14 (4.1%)	8 (8.1%)
B-receptor antagonists	248 (72.5%)	75 (75.8%)
ACE-inhibitors	132 (38.6%)	51 (51.5%)
ANG-II-antagonists	54 (15.8%)	13 (13.1%)
Ca^2+^-receptor antagonists	96 (28.1%)	36 (36.4%)
Short-acting nitrates	134 (39.2%)	43 (43.4%)
Long-acting nitrates	116 (33.9%)	52 (52.5%)
Aspirine	331 (96.8%)	90 (90.9%)
P2Y12-inhibitors	339 (99.1%)	81 (81.8%)

*CTO, chronic total occlusion; IVUS, intravascular ultrasound, ACE, angiotensin converting enzyme; ANG, angiotensine. Numbers are given as n (%) or mean ± SD.*

### Events

**FIGURE 2:**
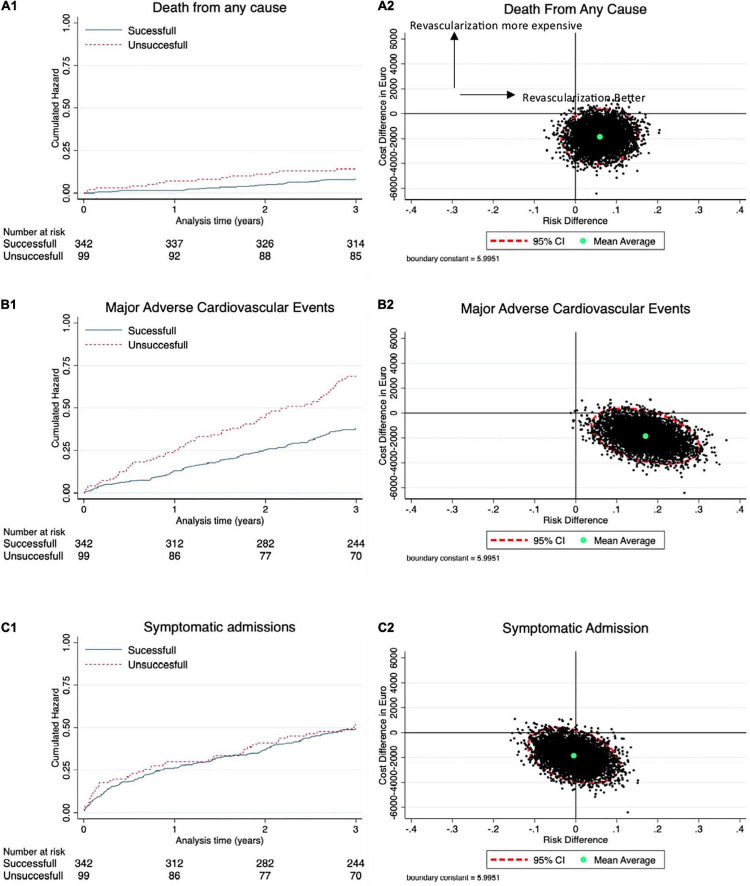
Cost effectiveness. **(A1)** Kaplan–Meier curves showing difference in all-cause death with corresponding cost-effectiveness (CE) plane in **(A2)**. **(B1)** Nelson–Aalen estimates of difference in the major adverse cardio- and cerebrovascular events (MACCE) with corresponding CE-plane in **(B2)**. **(C1)** Nelson–Aalen estimates of difference in symptomatic admissions with corresponding CE-plane in **(C2)**.

In the successful group, 8.2% of the patients died within 3 years compared with 14.1% in the unsuccessful group. About 21.3 and 38.4% of patients had at least one MACCE in the successful vs. unsuccessful group. In total, 155 MACCE events occurred, and 184 admissions due to suspected ACS occurred in the follow-up period with 24.3% of the patients in the successful group and 24.2% in the unsuccessful group having at least one event (Table 3). The Kaplan–Meier and Nelson–Aalen cumulative estimates are shown in Figure 2.

**TABLE 3:** Events 3 years after inclusion.

	Successful *n* = 342		Unsuccessful *n* = 99	
	
	Events	Patients with atleast one event	Events	Patients with atleast one event
MACCE	103	73 (21.3%)	52	38 (38.4%)
Death		28 (8.2%)		14 (14.1%)
Myocardial infarction	15	14 (4.1%)	7	7 (7.1%)
Stroke	5	5 (1.5%)	3	2 (2.0%)
Hospitalization for heart failure	22	16 (4.7%)	9	8 (8.1%)
Target vessel revascularization	33	27 (7.9%)	19	16 (16.1%)
Symptomatic admission				
Observation for MI	145	83 (24.3%)	39	24 (24.2%)
In hospital complications	30	23 (6.7%)	10	7 (7.1%)

*MACCE, major adverse cardiovascular and cerebrovascular events; MI, myocardial infarction. Numbers are given as n (%).*

### Cost Analysis

**FIGURE 3:**
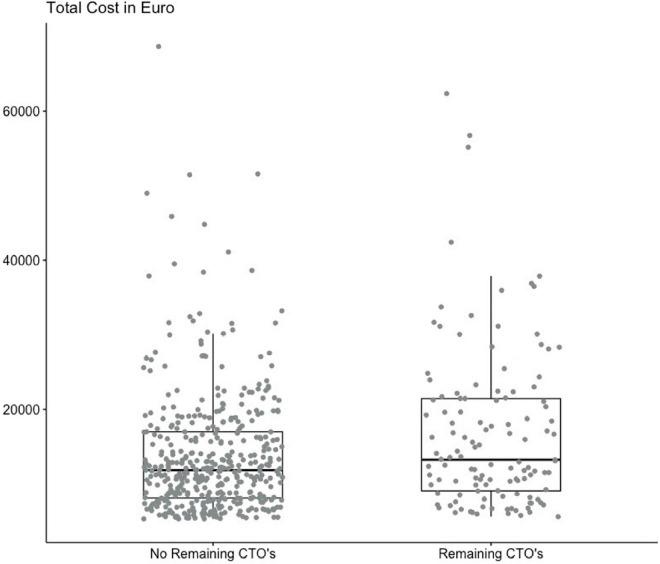
Cost distribution. Boxplot showing the distribution of costs for all individual observations with median and interquartile range (IQR) stratified on successful and unsuccessful revascularization.

The mean total cost was EUR 11.719 (11.034; 12.406) in the successful group vs. EUR 13.565 (11.899; 15,231) (*p* = 0.02) in the unsuccessful group, after 3 years of treatment. The primary endpoint of NB was EUR 1.846 (64; 3,627) (*p* = 0.02) after successful revascularization. Multiple linear regression adjusting for age, sex, LVEF, three-vessel-disease, and chronic kidney disease, which we have previously found to be possible confounders in the registry, found an adjusted estimate of NB to EUR 1,481 (–118; 3,079). The mean ICER for MACCE was –10.831 at 3 years (Table 4). Cost distribution in the two groups is plotted in Figure 3. CE-planes showed favorable cost with most bootstrap estimates lying in the south east (SE) quadrant for MACCE and death, and in the SE and south west (SW) quadrant for admission due to ACS (Figure 2). Furthermore, favorable effectiveness estimates were observed for death and MACCE in CE-plane analysis. For a cohort of 1,000 patients, annual event costs were EUR 226.635 [0; 1.00–1.586] in the successful group and EUR 395.730 [0; 1.16–3.942] in the unsuccessful group (*p* = 0.16).

**TABLE 4:** Index and event cost.

	Successful (*n* = 342)	Unsuccessful (*n* = 99)	Net benefit
**Procedural cost in Euro**			
Total cost			
Mean	9.429 ± 4.006	10.015 ± 4.538	584 (–423; 1,592)
Median	7.887 [6.496; 11.827]	8.164 [6,287; 11,579]	
Utensil’s cost	2.528 ± 1.925	2.355 ± 1.736	
Hospital cost	6,749 ± 2.973	7.477 ± 3.955	
Complication cost*	1,672 ± 3,669	2,502 ± 4,194	
**Event cost in Euro**			
Mean	2.289 ± 4.966	3.550 ± 7.230	1,260 (–253; 2,775)
Median	0 [0; 2.399]	0 [0; 4.854]	
**Total cost at 3 years in Euro**			
Mean	11.719 (11.034; 12.406)	13.565 (11.899; 15,231)	1,845 (64; 3,627)
Median	10.617 [6.769; 13,803]	11.191 [7.322; 17.587]	

ICER, incremental cost effectiveness ratio. All estimates are given as price in Euro per patient in mean (95% CI), mean ± SD or median [interquartile range, IQR]. *Complication costs are only calculated for patients with one or more complication(s).

### Sensitivity Analysis

Procedural success is defined as successful revascularization of the index. CTO showed similar CE-plane results (Supplementary Figure 1). The CE-planes derived from median costs showed similar results as those reported in the study (Supplementary Figure 2).

## Discussion

The present cohort study investigating cost-effectiveness after PCI of patients with CTO found that patients who were fully revascularized for all CTO lesions had both a more effective but less expensive treatment. Adjusted analysis found a small reduction in NB after adjustment for age, sex, LVEF, three-vessel-disease, and chronic kidney disease. Improvement in NB was persistent for symptomatic admissions and deaths but not statistically significant. The main findings underline the importance of having a high success rate when embarking on a CTO program, since this may lead to a more efficient but also less costly outcome for the patients.

The mean index cost was EUR 9.429 ± 4.006 and 10.015 ± 4.538, respectively, in the two groups. These numbers are comparable, yet a bit lower, than those previously found in the OPEN-CTO registry, where the overall index hospitalization cost was EUR 15.091 (converted from 17.048 USD in the original article) (9). Additionally, when considering the findings in the current study cover 1.4 ± 0.6 and 1.5 ± 0.8 procedures. This is probably due to the fact that the costs in the current study are based on average costs for PCI in Denmark, as well as the fact that healthcare cost rates are more expensive in the United States than in Denmark (14). The lower costs are also supported by a *post hoc* analysis using Markov modeling of the FACTOR trial where the index CTO treatment was EUR 6,639 ± 3,249 (15). In the present study, the increased cost of CTO PCI compared with non-CTO PCI were corrected by adding the cost of the utensils used at the procedure, since these are not incorporated into the national “DRG” rate. This is supported by the findings of Karmpaliotis and colleagues who found that the total cost is higher in patients treated with PCI for a CTO lesion compared with non-CTO lesions (16).

The present study is the first study to investigate the long-term cost-efficiency of patients with CTO undergoing PCI. However, it is important to underline that the findings do not support if revascularization is preferable compared with optimal medical therapy as investigated in the ORBITA cost benefit sub study (8). All patients enrolled in the current analysis had clinical indication for revascularization. Therefore, the results indicate that to improve the cost-effectiveness of revascularization, it is important to have high success rate to both improve prognosis and lower average costs. This underlines the importance of having a dedicated CTO program with high volume operators to increase the success rate as observed by Young and colleagues (17). However, as we have seen with several prediction models, operator expertise is not always sufficient in highly complex lesions (18), and therefore, it is also important to point out that preprocedural planning and especially abstaining from or referral of a complex CTO case is important for both to decrease the expenses on the healthcare system and also improve the success rate for complex cases. In low volume sites, very low success rates of < 50% are observed (19). Therefore, we argue that the interpretation of the data in the present study underlines the global consensus reached by CTO-operators from 50 countries that CTO-operators must be able to handle many different techniques and scenarios to optimize the success rate (20). A collection of CTO procedures at fewer sites will probably facilitate a higher success rate without an increase in complication rate (17, 19). The present study did not investigate if a more aggressive technique was beneficial, and the treating physicians must always outweigh the potential complications and benefits. Therefore, it is important to assure the indication (OMT resistant symptoms and reversible ischemia) before embarking on CTO-PCI. By extrapolating the NB in the current study, an increase in success rate of 10% in a cohort of 1,000 patients would decrease the cost of EUR 198.465. However, these data are merely hypothesis generating and must be confirmed in a prospective randomized trial. Success rate in the current registry is comparable with other similar registries, such as PROGRESS-CTO, OPEN-CTO, RECHARGE registry, EUROCTO-registry, and jCTO registry with success rates ranging from 85 to 89% (20). Furthermore, a high jCTO score was observed in the current study (3.1 ± 1.2 and 3.4 ± 1.0). A recent external validation of the jCTO score found that lesions with jCTO scores of 3 have a predicted success rate of 73.3% and an observed success rate of 72.0% (18). We acknowledge that even higher success rates may be attainable by selecting cases with less severe complexity and by future improvement of techniques.

Patients in the current study are true all-comers since we investigated all patients who underwent CTO PCI at AUH and registered in the EUROCTO database in a 10-year period. Patient characteristics are comparable to *all* patients diagnosed with a CTO in Sweden between 2005 and 2012, though a lower frequency of patients with three vessel disease was observed in the successful arm (11). It is worth mentioning that patients enrolled in prospective trials are less diseased than what was observed in our cohort (2, 21). Despite being comparable, differences between the groups in the study exists, and therefore, adjusting for clinical important factors found to be of statistical significance in a backward selection model was performed. The model included age, sex, LVEF, chronic kidney disease, and three-vessel-disease. A reduction in NB of EUR 365 was found. This indicates that only minor differences between the groups were confounding the results, however, residual confound may still be present.

The majority of studies looking into outcomes following CTO PCI investigates the primary exposure as successful vs. unsuccessful index procedure defined as the success of opening the CTO of interest during one procedure. In the present trial, we choose that the patients should be fully revascularized in all their CTO lesions because we hypothesize that this will lower the myocardium at risk. However, we performed a sensitivity analysis to investigate this matter and found a lower NB (NB = 821 [–1,124; 2,766]) (Supplementary Figure 1).

### Limitations

The current study is a retrospective analysis of prospectively collected data. Therefore, the data were collected for another intent than what was used in the current study, which may lead to a selection bias. Ischemic testing was not performed in routine clinical practice in the first part of the study period and these data are therefore not included. Only patients at one single center were included, compromising the external validity, however, the center is the only center performing PCI within the central region of Denmark, inhabiting 1.3 million citizens and covering all layers of society. Furthermore, 100–120 CTO cases are performed at the center each year, which may be a bit low compared with other very high-volume centers. However, Young et al. and Zein et al. observed that superior outcomes were found if operators had performed > 60 and 35 cases in total, respectively. In the study by Zein et al. only 4 sites (8.7%) performed > 50 CTO PCIs’ per year, however, we acknowledge that other very high-volume sites may be performing more procedures per year. We only evaluated events requiring hospital admissions, and therefore, the cost may have been underestimated in both groups. Furthermore, only events that occurred within the central region of Denmark were collected, and therefore a risk of underestimating event rate in both groups is present. We used DRG rates for the discharge diagnosis, and therefore, an underestimation of the cost may be present; however, this is consistent in both groups. No control group being treated with OMT alone was included. Patients treated with OMT alone for a CTO may have a significantly lower cost compared with PCI. This is supported by the findings in this study where two-thirds of the total cost at patient-level was contributed by the procedure(s). On the other hand, CTO-PCI may decrease the amount of anti-ischemic drugs used and therefore further lower costs in the successful arm, but the cost of medications was not captured in this trial. The findings in the present trial does not support the notion that successful revascularization of patients with CTO is more cost-efficient than OMT alone.

## Conclusion

We found that patients fully revascularized for all their chronic total occluded coronary artery lesion(s) compared with patients where the procedure failed had a more cost-efficient treatment. Fully revascularized patients both experienced a lower event rate and NB, confirming that the treatment is neither harmful nor more expensive. However, future prospective trials investigating outcomes after CTO PCI should focus on selecting the right patients for revascularization and thereby further increasing cost-effectiveness.

## Data Availability Statement

The datasets presented in this article are not readily available because according to Danish law we are not able to share data unless a data processor/exchange agreement is made. Requests to access the datasets should be directed to EH, eh@clin.au.dk.

## Ethics Statement

Ethical review and approval was not required for the study on human participants in accordance with the local legislation and institutional requirements. Written informed consent for participation was not required for this study in accordance with the national legislation and the institutional requirements.

## Author Contributions

EH and NW were responsible for data collection, data management, and analysis. EH and LM were responsible for statistical analysis. EH, LM, and EC were responsible for conceptualization and design of the study. EH drafted the manuscript. All authors critically revised the manuscript and approved the final version.

## Conflict of Interest

EH and EC received institutional funds from Asahi Corp., Phillips Corp., and Orbus Neich Corp. The remaining authors declare that the research was conducted in the absence of any commercial or financial relationships that could be construed as a potential conflict of interest.

## Publisher’s Note

All claims expressed in this article are solely those of the authors and do not necessarily represent those of their affiliated organizations, or those of the publisher, the editors and the reviewers. Any product that may be evaluated in this article, or claim that may be made by its manufacturer, is not guaranteed or endorsed by the publisher.
