# Supplementary material for: Cost-Effectiveness in Patients Undergoing Revascularization of Chronic Total Occluded Coronary Arteries—A Cohort Study
Source: Front Cardiovasc Med. 2022 May 26;9:849942. doi: 10.3389/fcvm.2022.849942 (PMC9177990; doi:10.3389/fcvm.2022.849942)

**Supplementary Material: Cost-Effectiveness in patients undergoing revascularization of chronic total occluded coronary arteries – a cohort study**

Supplementary Table 1: In hospital complications

|  | Successful | Unsuccessful |
| --- | --- | --- |
|  |  |  |
| Death | 0 (0.0%) | 1 (1.0%) |
| Acute renal failure | 13 (3.8%) | 4 (4.0%) |
| Stent thrombosis | 1 (0.3%) | 0 (0.0%) |
| Donor vessel dissection | 1 (0.3%) | 0 (0.0%) |
| Perforation | 7 (2.0%) | 2 (2.0%) |
| Vascular complication | 2 (0.6%) | 2 (2.0%) |
| Stroke | 0 (0.0%) | 0 (0.0%) |
| Resucitated Cardiac arrest | 4 (1.2%) | 1 (1.0%) |
| Procedural MI | 2 (0.6%) | 0 (0.0%) |
| Repeat revascularization | 0 (0.0%) | 0 (0.0%) |

Supplementary Figure 1: Cost-Effectiveness plane sensitivity analysis successful revascularisation versus unsuccessful of index CTO


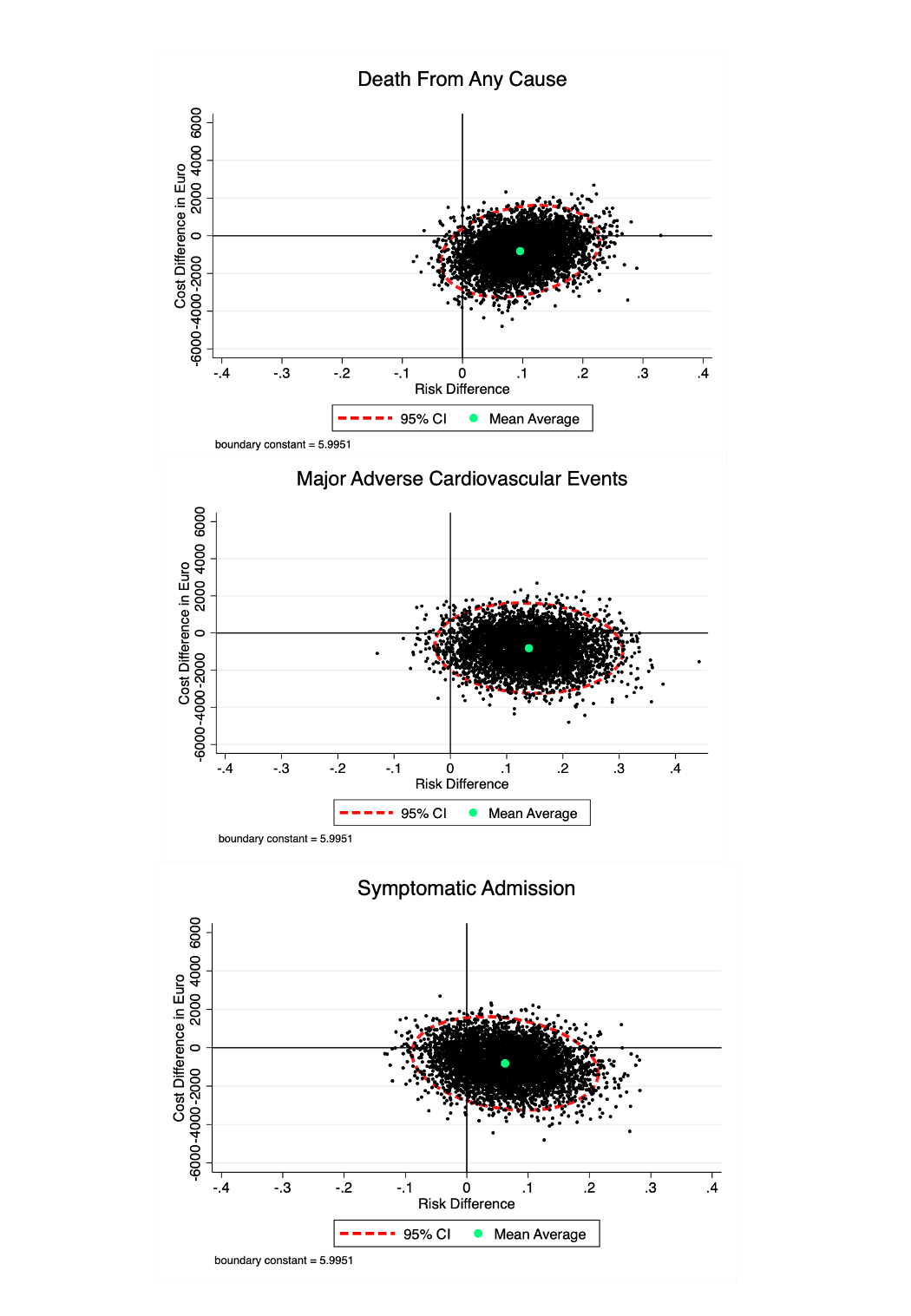


Supplementary Figure 2: Cost-Effectiveness plane sensitivity analysis median cost


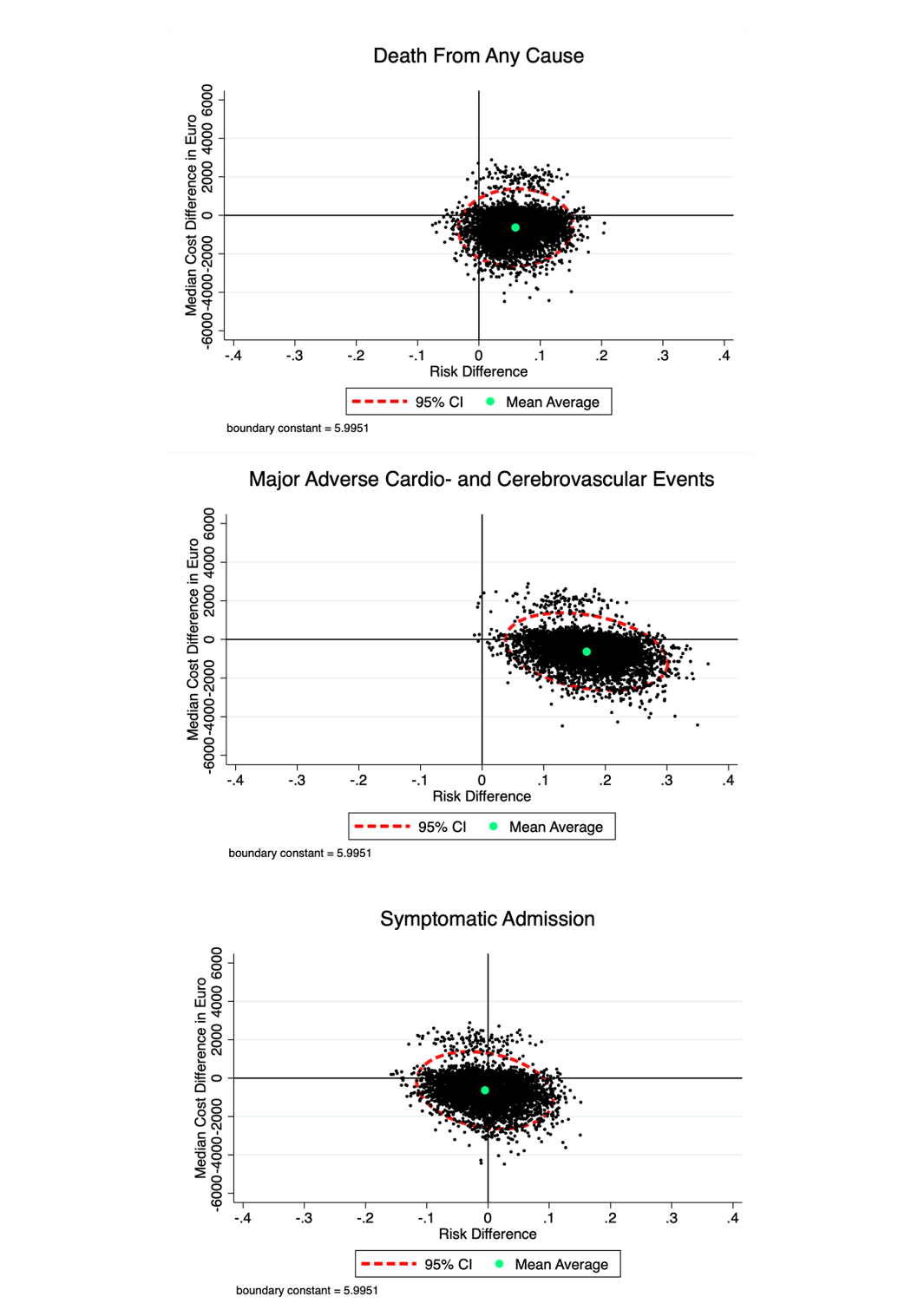

Supplement: Supplementary file 1 [file Data_Sheet_1.docx]
